# Alcohol policies in Malawi: inclusion of WHO “best buy” interventions and use of multi-sectoral action

**DOI:** 10.1186/s12889-018-5833-7

**Published:** 2018-08-15

**Authors:** Beatrice L. Matanje Mwagomba, Misheck J. Nkhata, Alex Baldacchino, Jennifer Wisdom, Bagrey Ngwira

**Affiliations:** 1 Lighthouse Trust, Lilongwe, Malawi; 2 School of Public Health and Family Medicine, University of Malawi-College of Medicine, Blantyre, Malawi; 3 Population and Behavioural Sciences Division, School of Medicine, University of St Andrews, Fife, Scotland; 4 Anthropology Department, Catholic University of Malawi, Chiradzulu, Malawi; 5 Department of Anthropology, Durham University, Durham, UK; 6 Wisdom Consulting, New York, USA; 7 Environmental Health Department, University of Malawi-Polytechnic, Blantyre, Malawi

**Keywords:** Harmful use of alcohol, Malawi, ANPPA, “Best buy” interventions, “Best-buys”, Multi-sectoral action, MSA, Policy analysis

## Abstract

**Background:**

Harmful use of alcohol is one of the most common risk factors for Non-Communicable Diseases and other health conditions such as injuries. World Health Organization has identified highly cost-effective interventions for reduction of alcohol consumption at population level, known as “best buy” interventions, which include tax increases, bans on alcohol advertising and restricted access to retailed alcohol. This paper describes the extent of inclusion of alcohol related “best buy” interventions in national policies and also describes the application of multi-sectoral action in the development of alcohol policies in Malawi.

**Methods:**

The study was part of a multi-country research project on Analysis of Non-Communicable Disease Preventive Policies in Africa, which applied a qualitative case study design. Data were collected from thirty-two key informants through interviews. A review of twelve national policy documents that relate to control of harmful use of alcohol was also conducted. Transcripts were coded according to a predefined protocol followed by thematic content analysis.

**Results:**

Only three of the twelve national policy documents related to alcohol included at least one “best buy” intervention. Multi-Sectoral Action was only evident in the development process of the latest alcohol policy document, the National Alcohol Policy. Facilitators for multi-sectoral action for alcohol policy formulation included: structured leadership and collaboration, shared concern over the burden of harmful use of alcohol, advocacy efforts by local non-governmental organisations and availability of some dedicated funding. Perceived barriers included financial constraints, high personnel turnover in different government departments, role confusion between sectors and some interference from the alcohol industry.

**Conclusions:**

Malawi’s national legislations and policies have inadequate inclusion of the “best buy” interventions for control of harmful use of alcohol. Effective development and implementation of alcohol policies require structured organisation and collaboration of multi-sectoral actors. Sustainable financing mechanisms for the policy development and implementation processes should be considered; and the influence of the alcohol industry should be mitigated.

## Background

Harmful use of alcohol, which refers to a pattern of alcohol consumption that causes damage to health, physically or mentally, has been identified as one of the world’s leading health risks [1]. In 2012, about 3.3 million deaths, or 5.9% of all global deaths (7.6 and 4.0% among males and females respectively) were attributable to alcohol consumption [1]. There are well described gender and age differences associated with levels of alcohol consumption. Adolescents in Sub-Saharan African countries consume substantially less alcohol compared to North America and Europe [2].

In 2009, a population survey for selected NCDs and their risk factors was conducted in Malawi, following WHO’s STEPwise (STEPS) protocol [3]. It was found that 68.1% of Malawians aged 24 years or older were lifetime alcohol abstainers [3]. A 2012 field-based survey also found that 14.5% of Malawians (27.3% of men and 1.6% of women) aged 18 years or older drank alcohol in the 12 months preceding the survey [4]. However, among those who consume alcohol, 40.8% (51.4% of males and 13.1% of females) are heavy episodic drinkers and total per capita alcohol consumption (for persons aged 15 years and above) is estimated at 15.9 l of pure alcohol per person per year among males and 4.6 l among females [5]. The high abstention rate contrasted with high total consumption rate in Malawi suggests that those who are drinkers have risky drinking patterns.

The World Health Organization (WHO) advocates for the development of national policies with various interventions targeting reduction of harmful use of alcohol [6]. The interventions are prioritized for implementation based on such criteria as cost-effectiveness, fairness, feasibility of implementation, and political considerations. Those identified as highly cost-effective and feasible for low resource constrained countries are referred to as “best buy” interventions (“best buys”) [7]. Three alcohol harm reduction “best buys” include: increased taxes on alcohol, restricted access to retailed alcohol and enforcement of bans on alcohol advertising [7].

Addressing the burden of NCDs and their associated risk factors requires a ‘whole-of-government’ approach [8] and application of multi-sectoral action (MSA) [9]. MSA refers to actions that are undertaken by non-health sectors, with or without collaboration with the health sector, to attain health-related outcomes or to influence determinants of health [10]. In Malawi, MSA has been applied successfully in the prevention and care of HIV and AIDS [11], facilitated by a strong political will in mainstreaming HIV interventions in all sectors and at all levels. Application of the alcohol “best-buy” interventions also calls for MSA since regulation of taxes as well as control of sales and advertising is a mandate of other government agencies than the Ministry of Health. However, the extent to which non-health sectors are involved in the policies related to control of harmful use of alcohol and NCD prevention in Malawi and sub-Saharan Africa (SSA) in general, is not known [12].

This paper describes the inclusion of alcohol related “best-buy” interventions and the extent of MSA in national alcohol control policies in Malawi. It further presents the actors, facilitators and barriers to MSA for the Malawi National Alcohol Control Policy development process.

## Methods

This study was part of a multi-country research project that focused on analysis of NCDs prevention policies in six African Countries (ANPPA) [12] which applied a qualitative case study design [13]. Although the study analyzed all NCD risk factors, this paper is only reporting findings from the alcohol related policy analysis which was more comprehensive than all other NCD risk factor areas that were analysed for Malawi .

### Data collection procedures

Data were collected using key informant interviews and document reviews.

### Key informant interviews

The study used purposive and snow-balling techniques [14] for sampling key informants. Initial set of key informants were identified from the acknowledgement sections of policies that were reviewed. During the initial interviews, participants mentioned other key participants in the policy formulation and implementation process who were later included as key informants for the study. A deliberate effort was made to include key informants from both government and non-governmental institutions. In addition, key informants from in-country bilateral development partners working in the health sector were also interviewed. Where a staff member had moved since the policy development period an alternate representative from the concerned institution was interviewed.

Using semi structured interview guides, key informant interviews were conducted to explore an in-depth understanding of actor involvement and to establish policy formulation processes and implementation status of NCD related prevention policies. This paper focusses on processes for policies related to harmful use of alcohol as an NCD risk factor. Key informant interviews also sought to determine the extent of MSA use, the facilitators and challenges during formulation and/or implementation of policies that involved multi-sectoral actors. Within the study, we defined MSA as the involvement of institutions from any two or more sectors, one of which must include a government institution. The interviews were audio-recorded using digital recorders after obtaining consent and noting participant’s demographic data.

### Document reviews

Documents that were reviewed in this study included: alcohol related policies, strategies and action plans, regulations and laws that were established at national level. This review included most recent versions of the available documents in ether hard copy or electronic formats. The policy documents were identified based on the investigators’ prior knowledge and information obtained from key informant interviews. This process generated a comprehensive list of all the relevant documents regarding alcohol related documents in Malawi. Some hard copies of national policy documents were accessed from the Policy Unit of the Office of President and Cabinet, some through key informants and others by reaching out to relevant government officials. A small number of these documents were downloaded from government websites.

The aim of the document reviews was to describe the policy content, identify existing policy interventions and how they relate to the WHO “best buys” for reduction of harmful use of alcohol. After the investigators had accessed the available policy documents, a Masters’ level research assistant was engaged to assist with extraction of the necessary information from the documents using a pre-designed template. The template was based on study objectives an items based on the Walt and Gilson’s policy analysis framework [15] that focuses on context, content, process and actors.

### Data analysis

#### Key informant interview data

The recorded key informant interviews were transcribed verbatim by research assistants with previous experience in qualitative research methods who received specific training for this study. Transcribed interviews were saved in Microsoft word format in a password-protected computer. Data cleaning involved reading of the transcripts by the investigators and the qualitative research assistant to get familiar with the data as well as to identify incomplete sections, typographical errors and formatting errors prior to data coding and analysis.

The transcripts were then uploaded into NVivo 10 for data management and coding. A coding framework in line with the ANPPA project code book [12] was then used for analysis. Initially, each research assistant coded one transcript and then discussed it with the rest of the team in a data coding workshop. During the coding process, each of the two investigators double-coded 10% of all the transcripts to ensure consistency in coding. In the few instances where discrepancies emerged, the investigators discussed among themselves first, then with all coding team members, to reach consensus on how to proceed. This process ensured concordance in the coded data.

After completion of the coding process, emerging themes were identified and key quotes were selected to be included in the narrative of the report. Thematic content analysis was conducted. A preliminary analysis involved identifying the texts linked with each thematic content area. Secondary level analysis was done to further analyse the data by adding other emerging themes outside the established framework.

#### Document review data

We analysed alcohol related policy documents by examining the policy rationale (which highlighted the context in which the policy was formulated), year of development, objectives, planned interventions and whether there is an accompanying implementation plan or specific mechanisms through which the policy would be actualized. We assessed whether each of the policies included any intervention related to WHO “best buy” interventions for reduction of harmful use of alcohol. We further assessed if there was MSA at formulation by identifying actors from acknowledgement sections of each of the policy documents. However, the documents alone could not comprehensively show the roles and extent of involvement of all the actors. Hence, these findings were triangulated with information from the key informant interviews. In this study, MSA was considered non-existent if less than two sectors were involved, low if 2 to 4 sectors, medium if 5 to 9 sectors and high if 10 or more sectors were involved.

#### Data quality measures

To ensure that quality data were collected, we ensured all research staff had prior research experience. Interviewers underwent training in qualitative interviewing techniques, transcription and coding. Data collection tools were pretested and interviewers collected data in pairs and peer reviewed the transcribed pilot data for standardization and to increase accuracy. Any challenges encountered in data collection, framing of questions and areas for further probing were discussed before actual data collection.

The study protocol was reviewed and approved by Malawi’s National Heath Science and Research Committee. The study participants provided written informed consent.

## Results

### Participant characteristics

Thirty-two key informants were interviewed for this study. Their sector affiliation is summarized in Table 1. Nineteen of the key informants were drawn from government with seven from the Ministry of Health. Of the remaining thirteen key informants from non-governmental/non-state actors and international partner organizations, nine were from Non-Governmental Organizations (NGOs) and from NCD-related alliances and/or associations.

**Table 1:** Number of Key Informants by Sector

Respondent type	Total number
Government Actors	19
Health	7
Industry and Trade	1
Agriculture	2
Youth	2
Home Affairs	3
Labour	1
Office of President and Cabinet	1
Other Government sectors (Parastatal)	2
Non-Government/Non-State actors	
NGOs/Civil Society/Associations (health sector)	9
Research/Academic institutions (Agriculture sector)	1
International Organizations (health sector)	1
Bilateral Organizations (health sector)	1
Universities/ Research (non-health sector)	1
Total	32

### Alcohol policies for Malawi

We reviewed twelve national policy documents that relate to alcohol use control (See Table 2). These included five Acts of Parliament, one Government Regulation, four Strategic and /or Action Plans and two Policies.

**Table 2:** Documents Reviewed

Document Type	Document
Acts	Taxation Act 2001
	Road Traffic Act 1998
	Consumer Protection Act 2003
	Liquor Act, of 1979 (Last Amended in 2000) Chapter 50:07 of the Laws of Malawi
	Malawi Bureau of Standards Act 2012
Regulations	Liquor (Production, Marketing & Distribution) Regulation 2015
Strategic and/or Action Plans	National Road Safety Strategic and Five year Action Plan (2015–2020)
	National Drug Control Master Plan (2005)
	Health Sector Strategic Plan (2011–2016)
	National Action Plan for Prevention and Management of NCDs in Malawi (2012–2016)
Policy	National Health Promotion Policy (2013)
	National Alcohol Policy (2017)

### Inclusion of “best buy” interventions

Among the twelve documents that were reviewed, only three included at least one “best buy” intervention and this was mainly on restricted access to retailed alcohol (See Table 3). The three documents are: the Liquor Act, of 1979 (Last Amended in 2000) Chapter 50:07 of the Laws of Malawi, the 2015 Liquor Production, Marketing & Distribution Regulations, and the National Alcohol Policy (2017). None of the policy documents included “best buy” interventions related to increased taxation and ban on advertising.

**Table 3:** Inclusion of WHO “Best Buys” in Alcohol Policies in Malawi

Policy	Year of Development	Lead Sector	“best buys” addressed		
			Restricted access to the retailed alcohol	Tax increases	Bans on alcohol Advertising
Liquor Act, of 1979 (Last Amended in 2000) Chapter 50:07 of the Laws of Malawi	1979 (amended in 2001)	Local Government	Yes	No	No
Liquor (Production, Marketing & Distribution) Regulations	2015	Industry and Trade	Yes	No	No
The National Alcohol Policy	2015–2017	Health	Yes	Partial	Partial

All documents included other interventions outside the “best buys”, some of which are among the ten global policy options meant to control and reduce harmful use of alcohol [6]. The other globally recommended interventions identified during document reviews were: counter measures against drunk-driving, promotion of awareness of alcohol related harm and health system response with interventions targeting individuals who are at risk or already affected by harmful use of alcohol.

### Rationale and objectives of the current alcohol policies

Malawi’s Liquor Act, of 1979 [16] was developed to give powers to local governments to regulate the manufacture and sale of intoxicating liquor by granting permits/licenses to businesses intending to manufacture or sell beer and other alcoholic beverages. The Liquor Act covers restricted access to retailed alcohol by stipulating the permitted hours for the sale of liquor by permit holders who are approved by the Local Government. The Act also restricts the supply of opaque and traditional beer to young people by stipulating that “opaque or traditional beer shall not be supplied to persons under the age of 18 years” [16].

The 2015 Liquor Production, Marketing & Distribution Regulations [17] were developed to prohibit the manufacturing and importation of liquor packaged in plastics or polythene bags as well as the packaging of industrial grade ethanol for purposes of consumption. The regulations are also aimed at regulating the quantities and packaging standards of alcoholic beverages. The regulations intend to control the availability of cheap liquor to prevent people from consuming harmful quantities of liquor in Malawi. The regulations address the “best buy” interventions by imposing restrictive standards on production and packaging about the type of liquor, volume and package materials used.

The National Alcohol Policy was in final draft form (Cabinet Paper level) at the time of document analysis but was later approved by cabinet in January 2017 [18]. It is an overarching policy for controlling harmful use of alcohol in Malawi. It provides comprehensive mechanism for development, implementation, coordination, monitoring and evaluation of health, social and economic interventions related to harmful alcohol consumption in Malawi [18]. The main policy objective is to reduce the health and socioeconomic burden associated with harmful use of alcohol through strategies that include: ensuring effective regulation of availability and accessibility of commercial alcoholic products (including informally produced alcoholic products), promoting health and other sectors’ response to harmful use of alcohol, ensuring reduction in demand for alcoholic products through behavior change related interventions, and promotion of monitoring, surveillance and research on harmful use of alcohol. The policy advocates for regulation of tax for alcoholic products based on percentage of alcohol volume, albeit it does not indicate the tax increase schedule to be adopted. The policy further states regulation of advertising and marketing of alcoholic drinks as one of priority intervention areas. However, the policy implementation strategy does not include a total ban of alcohol advertising.

In the following section, we explore the formulation process and use of MSA for the National Alcohol Policy. We focus on the National Alcohol Policy because it was the most recently formulated policy(hence reducing possibilities of recall bias) but also because through its initial development processes, Malawi was previously hailed as a best practice in alcohol policy formulation [19].

### Formulation process and timelines for the National Alcohol Policy

The first step for establishing an alcohol policy in Malawi was initiated in 2006 by the alcohol industry. Several NGOs including Drug Fight Malawi, that were invited to the initial industry facilitated consultative meeting noted, with concern, that the aim of the policy was to encourage responsible drinking and that alcohol industry led policy had limited scope. There was also an absence of representation from government ministries responsible for alcohol harm control. As such, these NGOs abandoned the industry-led policy. By 2007, with support from a Norwegian-based international development organization, For Utvitling/Development (FORUT), Drug Fight Malawi initiated a new national policy formulation process with involvement of Malawi Government authorities and other stakeholders. National level consultation process for a comprehensive national alcohol policy started in 2008 [19].

By 2009, a multi-sectoral National Task Force was formed to develop the National Alcohol policy. The task force included relevant ministries and departments such as Ministry of Home Affairs and Internal Security, Ministry of Health, Ministry of Education, Ministry of Trade and Industry, Ministry of Youth, Ministry of Local Government, Ministry of Gender, Ministry of Information, Ministry of Justice, Malawi Police Service, National Youth Council; and members from various NGOs and Civil Society organizations. In line with WHO Global Alcohol Strategy [6], the alcohol industry was notably not included in the National Task Force. The National Task Force conducted consultative meetings in eight representative districts with local stakeholders including traditional leaders, religious leaders, local NGOs and the public. Apart from the National Task Force, a consultant was hired by FORUT to help with the zero-drafting of the policy. The drafts were reviewed by the task force in a series of meetings.

By June 2011, a national stakeholders’ meeting was held to validate the draft national alcohol policy. Among the attendees of the stakeholders’ meeting were representatives of the alcohol industry who formally raised concerns regarding their exclusion from the formulation process and that the awareness promotion component of the draft policy was weak because responsible drinking was not covered. This necessitated further review of the draft alcohol policy by the National Task Force, led by the NCD and Mental Health Unit of the Ministry of Health that had been nominated to take over coordination and finalisation of the policy.

The revised (pre-final draft) national alcohol policy had addressed some issues raised by the alcohol industry. However, after presenting it to the health sector Essential Health Package Technical Working Group (TWG) on 6th February 2013 [National NCD Program Manager, MOH, 2015], the TWG recommended that the responses to the concerns by the alcohol industry be presented to the alcohol industry representatives. Thus, on 7th March 2013, a formal consultative dialogue involving the Ministry of Health, members of the NTF and representatives of the alcohol industry was held [National NCD Program Manager, MOH, 2015].

Between mid-2013 and end-2014, the draft NAP underwent endorsement processes by the Senior Management Team at the Ministry of Health, inter-ministerial Principal Secretary’s committee, the Parliamentary Committee on Health and the Cabinet Committee on Social and Health. All these committees made inputs that were taken into consideration in finalising the National Alcohol Policy and writing a cabinet paper that was submitted to the full Cabinet in August 2016. Eventually, the National Alcohol Policy for Malawi was adopted in January 2017, eight years after the first national consultations.

### MSA in formulation of the National Alcohol Policy

Data from key informant interviews and a review of the national alcohol policy show that actors from several sectors were involved in its formulation. The extent of MSA was high since there were more than ten sectors involved. The sectors and their actors as well as their specific roles are presented in Table 4. The Government, through the Ministry of Health and the Ministry of Home Affairs had participated extensively in the formulation process. Similarly, CSOs and WHO had a significant level of involvement through financial and technical support to the process. Other members of the National Task Force participated in the meetings around formulation and are expected to lead in the implementation.

**Table 4:** Actors’ involvement and their roles

Sector	Actors	Type	Role in the policy process
Health	Ministry of Health	Government	Leading role in the development and implementation. Coordination of the process through the NCDs unit
	Drug Fight Malawi	CSO/Local NGO	Financial and technical support
	Malawi Alcohol Policy Alliance	CSO/Local NGO	Advocacy support
	JournAIDS	CSO/Local NGO	
	World Health Organisation (Malawi Office)	International Organisation	Technical support
	FORUT	International Organization	Financial and technical support
Law enforcement	Ministry of Home Affairs	Government	Initially led the formulation of the alcohol policy (2009–2011). Actively participated throughout the formulation and acts as a key enforcement agency
	Malawi Police Services	Government	Stakeholders involved as part of National Task Force during the formulation process. Actively participated in writing and review meetings
Industry and Trade	Ministry of Industry and Trade	Government	Stakeholders involved as part of National Task Force during the formulation process. Actively participated in writing and review meetings
	Alcohol industry	Private	
Communication/Information	Ministry of Information	Government	
	Electronic and Print Media Houses	Media	
Parastatals	National Youth Council		
	Malawi Bureau of Standards		
Other ministries	Office of the President and Cabinet	Government	Stakeholders involved as part of National Task Force during the formulation process. Actively participated in writing and review meetings
Other ministries	Ministry of Local Government	Government	
Other ministries	Ministry of Gender and Community Services	Government	
Justice	Ministry of Justice	Government	
Education	Ministry of Education	Government	
Other ministries	Ministry of Youth	Government	
Professional Association	Teachers Union of Malawi	CSO/Local NGO	Stakeholders involved as part of National Task Force during the formulation process. Actively participated in writing and review meetings
Academic/Research	Centre for Social Research, University of Malawi	Academic/Research	Evidence generation through Alcohol studies

### Facilitators for use of MSA in alcohol policy formulation

Several factors were identified as having facilitated use of MSA in the National Alcohol Policy formulation process. Firstly, government ministries led the formulation process which ensured that the policy was a national issue and hence improved participation of different sectors. From 2009 to 2011, the initial consultation and formulation process was led by the Ministry of Home Affairs and Internal Security with support from Drug Fight Malawi. From 2011, the Ministry of Health, through the Non-Communicable Diseases and Mental Health Unit, took over the coordination role in the National Alcohol Policy formulation process. Apart from the government leadership role, civil society support also facilitated the formulation process. Drug Fight Malawi, with funding from FORUT, was instrumental in the formulation of the Malawi National Alcohol Policy through financial support for consultative meetings, facilitation of meetings, technical support and advocacy. The formulation process was also supported by the WHO through provision of technical support.

Secondly, there was a National Task Force which included stakeholders who had a shared common understanding of the burden of harmful use of alcohol. Each of the stakeholders felt there was a need to formulate this policy.

*“I think basically we all saw that we had a common interest in terms of how the ministry of health presented the problem. Everyone saw that it was a serious issue [for example] how alcohol was being abused by children. So, the main reason was that everyone saw that this is serious. [They thought] let’s tackle this issue [and] develop a policy and after that maybe we can have a regulatory framework and some laws. So, the biggest factor was simply the seriousness of the problem.” (Industry Officer, Ministry of Industry and Trade)*

While all stakeholders were keen on developing an alcohol policy, their rationale was different. This was based on their varied perceptions of problems caused by harmful use of alcohol. The perceived problems included: negative impact on education especially among young people, road traffic accidents, increasing cases of gender based violence and increasing prevalence of NCDs.

Regular update meetings and communication among the stakeholders was also highlighted as a key factor for MSA in the formulation of the alcohol policy. This was made possible with limited funding.

*“There was back and forth communication between the regional office and the country office, and the other stakeholders at the national level, providing advice as to what extent we can we involve the [alcohol] industry. So those were some of the key guiding [principles]… [We used] those guidelines to make sure that the process of developing the policy was meeting the public health objectives that it was intended for.” (Health Promotion Officer, International NGO)*

### Barriers for use of MSA in policy formulation

Key informants identified several challenges in involving different stakeholders in the formulation of the alcohol policy. The first challenge to involving several sectors was funding. There was limited funding to involve different stakeholders in meetings and workshops for the formulation of the alcohol policy in Malawi.

*“I think costs for conducting meetings. The NCD unit sometimes did not have the funding, probably that also could be the reason why they couldn’t have so many people.” (Nutrition Officer, Ministry of Health)*

*“Well I think one of the challenges is resources to try and get opinions of different stakeholders on board” (Health Project Coordinator, International NGO)*

The second barrier was high staff turnover rates through transfers within and between departments/ministries, resignations and/or retirement. Some sectors were represented by different people at different meetings and workshops. In some cases, there was no proper handover of what was to be discussed at subsequent meetings.

*“The major challenge was coordination. If you are having different sectors, the first thing is it’s not possible to have consistent participation of the members. So, you keep on having new members coming in [because] some members have been transferred. Some sectors like education, some NGOs and (…) civil societies like Teachers Association of Malawi would have permanent members. Sectors like home affairs still have the same people who have been in the process from that beginning to the present, but other sectors like Ministry of Trade would have a senior person coming [for one meeting] and then a junior person coming in [for a subsequent meeting]. That, sometimes caused problems.” (Health Promotion Officer, International NGO)*

The third barrier was coordinating the different sectors and getting their views and interests on board. The variety of sectors meant that some of their views were very different and it was difficult to include all of them on board. Differences in views and interests among sectors was also a source of conflict in the formulation of the alcohol policy. Initially, there was competition as to who would lead the process between the Ministry of Health and the Ministry of Trade and Industry. There was also conflict due to differences in mandates and objectives. While the Ministry of Health emphasized the public health effects of alcohol, the Ministry of Trade and Industry was also interested in alcohol as a source of revenue and economic development of the country.

The fourth barrier to MSA was interference by the alcohol industry. Initially, the alcohol industry had initiated an alcohol policy process based on self-regulation. Even after the NGOs abandoned the initial process and alerted government on the need for a national alcohol policy that is independent of alcohol industry interests, the industry still took part in some of the discussions on the formulation of the alcohol policy through dialogue sessions. During these meetings, alcohol industry representatives were reported to have facts that countered what government and NGOs were presenting, specifically highlighting that alcohol is not as harmful as the NGOs were stating. Some key informants also suggested that the alcohol industry was reported to have approached some members involved in the alcohol policy formulation to influence them to support the views and perspectives of the industry.

*“When you look at the alcohol industry, the way I’ve seen them behaving is that they will make direct contact with those who are trying to formulate a policy or the different key ministries that are stakeholders. They will also approach the ministers and give them a reason why alcohol is important to Malawi. They give very convincing reasons [for example], taxes [from sale of alcohol] and their effects on the budget. But they don’t really talk about the health and social impact of alcohol. They go to influential leaders of the society to try and get them on their side and maybe to try and reduce the progress of the alcohol policy rolling out. That’s one of the things they do.” (Health Project Coordinator, International NGO)*

Despite the barriers, Malawi achieved a multi-sectoral process to National Alcohol Policy formulation. The policy stipulates similar coordinated efforts that have been planned through establishment of multiple multi-sectoral structures at every implementation level [18]. However, these barriers ought to be mitigated to effectively achieve MSA through the implementation of the National Alcohol Policy.

## Discussion

This section presents discussions around broad themes that represent the findings of this study and it covers: alcohol policy availability and content; the role of non-state actors including the alcohol industry; the enabling and challenging factors for use of MSA in alcohol policy processes; and limitations faced by this study that should be considered when translating the findings.

### Availability and content of alcohol polices in Malawi

Malawi has policies in place aimed at reduction of harmful use of alcohol. Interestingly, among the three policies that included WHO “best buy” interventions, only one intervention was fully addressed: the restricted access to retailed alcohol. Although most interventions addressed are according to the WHO Global Strategy for reduction of harmful use of alcohol [6], the “best buys” were not adequately prioritised. Specifically, increased taxes on alcohol and enforcement of bans on alcohol advertising have not been directly addressed by the National Alcohol Policy.

Several factors could explain the partial inclusion of “best buy” interventions in the National Alcohol Policy. In part, this could be explained by the fact that the policy was initiated prior to the introduction of the WHO “best buys” interventions in 2011. This could also be attributed to lack of knowledge and evidence of the “best buy” interventions among the policy makers in Malawi. Another explanation could be lack of political will to implement “best buy” interventions that may bring conflict with the alcohol industry such as increasing taxes on alcohol. Related to the lack of political will are the competing interests by different government departments in terms of balancing public health needs and the need to maintain alcohol manufacturers and traders as a source of revenue to the national economy. Additionally, interference by the Alcohol Industry during the policy formulation process might have diluted the content of the policy.

Despite the partial inclusion of “best buy” interventions, the National Alcohol Policy stipulates the need to revise various laws that address the alcohol related interventions by various government departments and agencies. Through such revisions, there are opportunities that “best buy” interventions could be included in policies and laws related to alcohol production and consumption. This is similar to a 2010 finding in South Africa whereby alcohol policy development took place in a piecemeal fashion with different pieces of regulations all aimed at control of harmful use of alcohol [20]. However, having a comprehensive national alcohol regulation was recommended as a better way to proceed [20]. Therefore, for effective implementation of a comprehensive national alcohol policy, Malawi might consider revising the Liquor Act to reflect the policy interventions relevant for comprehensive control of harmful use of alcohol. The current Liquor Act (1979) focuses on restriction of retail sales of liquor through licensing, but falls short of the other “best buy” interventions for alcohol harm control.

### The role of non-state actors in alcohol policy formulation process

This study has shown that MSA was extensively used in the formulation of the National Alcohol Policy. This is in keeping with previous appraisal of Malawi’s alcohol formulation process as a best practice [19]. During the formulation process, non-state actors played important facilitation roles. Among other roles, some NGO and CSOs provided funding and technical support for the alcohol policy formulation process.

Although the Malawi’s alcohol policy formulation had a commendable use of MSA, the process was very protracted lasting 8 years between establishment of the multi-sectoral National Alcohol Task Force (2009) and adoption of the National Alcohol Policy (2017). The protraction is largely attributable to interference by the industry as well as to dependence on NGOs for financial support to enhance the process. The NGOs and CSOs played a role in ensuring that the national alcohol policy formulation was not facilitated or fully seized by the alcohol industry to advance their interests.

### The role of the alcohol industry

Although it is already endorsed that there is no role for the global alcohol industry in the formulation of public health policies [21], he industry’s involvement from beginning of alcohol policy formulation processes is evident in at least four sub-Saharan African countries including Lesotho, Uganda, Botswana and Malawi [22]. Although Malawi moved on to disregard the first national policy draft that was facilitated by the industry, the process to the final National Alcohol Policy [18] still had some interference by the alcohol industry as shown by the finding that the industry demanded for a revision of the draft alcohol policy in their written complaint to the Government.

While the active involvement of the alcohol industry ought to be avoided during policy formulation [21], the industry plays important role during implementation stage of the policy. Without the industry’s collaboration, some of the policy interventions may not be effectively implemented such as ban of alcohol advertising. Alcohol adverts often accompany sponsorships for important public events and its ban might requires a strong political will and positive collaboration from the industry. Therefore, inclusion of the industry as member of Malawi’s National Committee on Alcohol [18] is an important mitigation measure against total sabotage of the policy. However, the industry contribution in such a committee ought to be objectively kept under control.

### Enabling factors for MSA in alcohol policy formulation

Several factors that facilitated the use of MSA in the alcohol policy formulation. The leadership from a government line ministry and a supporting NGO (Drug Fight Malawi) as well as having a structured coordination mechanism (a national task force) were among key factors for Malawi’s stakeholder involvement. Presence of a national multi-sectoral coordination mechanism has already been proposed as one of essential elements for effective MSA [23]. If the structured coordination mechanism demonstrated at policy formulation stage continues at implementation of the adopted National Alcohol Policy, Malawi is likely to have effective use of MSA for control of alcohol-related harms. Other perceived facilitators included: shared appreciation of the seriousness of the problem among various actors from different sectors, availability of funding for the process, regular updates through meetings and other forms of communication. These perceived factors ought to continue through implementation in order to maintain active stakeholder involvement and to effectively achieve the goals of the National Alcohol Policy.

### Challenging factors for use of MSA in alcohol policy formulation

The study has revealed that limited or inadequate funding for policy formulation, difficulties emanating from coordinating different sectors with different interests, lack of continuity due to high turnover of key personnel from relevant sectors and the influence of the alcohol industry are hindrances for MSA in the alcohol policy formulation process.

Funding for the policy formulation process was dependent on funding from NGOs. This is because there is lack of sustainable national financing mechanisms for NCD prevention policies including alcohol policies. Without such financing mechanisms, coordinating structures for MSA cannot function adequately.

Competing interests among various actors is another barrier in using MSA in policy formulation. We have noted above the differences in interests of the Ministry of Health and the Ministry of Industry and Trade. One of the strategies to deal with such competing interests among actors is to exclude those with competing interests for example those from alcohol and tobacco industries as advocated by the WHO [10]. Our study has revealed that such competing interests can exist even between ministries that are key to the formulation and implementation of a policy. In such cases, balancing these interests may be very important to ensure MSA in the policy formulation process.

### Study limitations

As this study was a retrospective analysis of policy development, some of the policies that were analyzed had been formulated long before the study was conducted. As such, it was difficult to get participants who were involved in the formulation of these policies. Further to this, participants had problems recalling all the processes involved in the formulation.

Some of the documents that were meant to be analyzed were not readily available from participants and government ministries responsible for policies. As such, this limited our analysis of multi-sectoral action in the formulation of the policies. While we could mention the sectors that were involved, it was not possible to find objectively verifiable evidence for actual contributions of the actors during the formulation process.

## Conclusions

Malawi’s national legislations and policies have inadequate inclusion of the “best buy” interventions for control of harmful use of alcohol. WHO should ensure that there is knowledge and evidence of the “best buy” interventions to both policy makers and technical assistants in all its member states. This could help to reinforce inclusion of the “best buys” in the legislations and policies and implementation of strategies that can contribute to the control of harmful use of alcohol.

Effective MSA in development and implementation of alcohol policies requires structured organisation; collaboration of the multi-sectoral actors; and sustainable financing mechanisms for both development and implementation processes. Consideration of the industry demands during policy formulation can derail the process as well as hinder full inclusion of some key interventions. Policy making stakeholders should be aware of the subtle interference mechanisms by the industry and should devise ways of mitigating or overcoming such interference at all stages of the policy process.

## Abbreviations

ANPPA: Analysis of Non-communicable Disease Prevention Policies in Africa
CSO: Civil Society Organisation
MSA: Multisectoral Action
NGO: Non-governmental Organisation
NTF: National Task Force
WHO: World Health Organization
